# Signs and symptoms of acute mania: a factor analysis

**DOI:** 10.1186/1471-244X-11-137

**Published:** 2011-08-19

**Authors:** Raveen Hanwella, Varuni A de Silva

**Affiliations:** 1 Department of Psychological Medicine, Faculty of Medicine, University of Colombo, Sri Lanka

## Abstract

**Background:**

The major diagnostic classifications consider mania as a uni-dimensional illness. Factor analytic studies of acute mania are fewer compared to schizophrenia and depression. Evidence from factor analysis suggests more categories or subtypes than what is included in the classification systems. Studies have found that these factors can predict differences in treatment response and prognosis.

**Methods:**

The sample included 131 patients consecutively admitted to an acute psychiatry unit over a period of one year. It included 76 (58%) males. The mean age was 44.05 years (SD = 15.6). Patients met International Classification of Diseases-10 (ICD-10) clinical diagnostic criteria for a manic episode. Patients with a diagnosis of mixed bipolar affective disorder were excluded. Participants were evaluated using the Young Mania Rating Scale (YMRS). Exploratory factor analysis (principal component analysis) was carried out and factors with an eigenvalue > 1 were retained. The significance level for interpretation of factor loadings was 0.40. The unrotated component matrix identified five factors. Oblique rotation was then carried out to identify three factors which were clinically meaningful.

**Results:**

Unrotated principal component analysis extracted five factors. These five factors explained 65.36% of the total variance. Oblique rotation extracted 3 factors. Factor 1 corresponding to 'irritable mania' had significant loadings of irritability, increased motor activity/energy and disruptive aggressive behaviour. Factor 2 corresponding to 'elated mania' had significant loadings of elevated mood, language abnormalities/thought disorder, increased sexual interest and poor insight. Factor 3 corresponding to 'psychotic mania' had significant loadings of abnormalities in thought content, appearance, poor sleep and speech abnormalities.

**Conclusions:**

Our findings identified three clinically meaningful factors corresponding to 'elated mania', 'irritable mania' and 'psychotic mania'. These findings support the multidimensional nature of manic symptoms. Further evidence is needed to support the existence of corresponding clinical subtypes.

## Background

The International Classification of Diseases-10 (ICD-10) and Diagnostic and Statistical Manual-IV (DSM-IV) classifications consider mania as a uni-dimensional illness [1,2]. Both systems classify bipolar illness as distinct categories of hypomania, mania, mania with psychotic features and mixed episodes. While categorical diagnosis used in ICD-10 and DSM-IV describe distinct symptom categories, a multi-dimensional classification of mania describes a continuing variation in symptoms [3].

Evidence from factor analysis suggests more categories or subtypes than those included in the ICD-10 and DSM-IV classification systems. The earliest factor analytic study on a small sample of 12 patients with mania by Beigel and Murphy proposed two factors, 'paranoid-destructive' and 'euphoric-grandiose' [4]. Subsequent studies have found a greater number of factors [5-7].

Double performed a cluster analysis of 81 manic patients using the Young Mania Rating Scale (YMRS) and described three factors: thought disturbance, overactive and aggressive behaviour, and elevated mood and vegetative symptoms. In this study principal component analysis separated elation from aggressiveness [6]. Dilsaver et al. using the Schedule for Affective Disorders and Schizophrenia in 105 patients identified four factors corresponding to manic activation, depressed state, sleep disturbance, and irritability/paranoia. Their findings suggest that manic episodes can be classified as classic (predominately euphoric), dysphoric, or depressed [8]. Picardi et al. using the Brief Psychiatric Rating Scale on 88 manic patients identified four factors: mania, disorganization, positive symptoms and dysphoria. The authors suggest the possibility of separating manic patients into two groups based on the presence of disorganization symptoms and investigating if these groups respond differently to treatment [9]. Rossi et al. analysed a sample of 146 patients with mania and mixed mania and identified five factors corresponding to activation-euphoric, depressive, psychomotor retardation, hostility-destructive and sleep disturbances [10].

While factors corresponding to euphoria, aggressiveness and irritability have been identified in most studies, some also report a depressive/dysphoric factor. A depressive factor has been identified in studies which used rating scales that incorporate depressive symptoms. Some of these studies also included patients with mixed mania. This depressive/dysphoric factor includes variables such as depressed mood, suicidal ideation and guilt [5,8,11].

Identifying different factor structures using factor analysis has clinical implications. Houston et al. showed that factors for 'psychomotor activity' (YMRS items for elevated mood, increased motor activity, and increased speech and the Hamilton Depression Rating Scale-21 (HDRS-21) agitation item) and guilt/suicidality (HDRS-21 items for guilt and suicidality) significantly predicted endpoint remission in patients treated with divalproex and olanzapine [12]. Patients with even marginally high guilt/suicidality were less likely to remit than those with lower levels of symptomatology. Swann et al. demonstrated that different types of symptom clusters are associated with different demographic characteristics and prognosis [13].

Although factor analytic studies have been used extensively in interpreting the phenomenology of schizophrenia and depressive disorder, studies of manic episodes are few. Some factors identified in these studies are not clinically meaningful. The present study includes a substantial sample of manic patients and we explored the hypothesis that the pattern of symptoms in manic episodes consist of a multi-dimensional structure which could be used to support distinct clinical subtypes. The findings of this study and similar factor analytical studies can lend empirical support for changes to classification systems such as the Diagnostic and Statistical Manual-IV (DSM-IV) that are being undertaken currently.

## Methods

The sample included 131 patients, consecutively admitted to an acute psychiatry unit with a diagnosis of manic episode, over a period of one year. Of the patients who fulfilled selection criteria six were not enrolled as they were too disturbed. There were no dropouts.

Written informed consent was obtained from all participants. No payment was made for participation. Ethical clearance for the study was obtained from the Ethics Committee of the National Hospital of Sri Lanka.

Patients were assessed independently by a trainee psychiatrist and a psychiatrist using a standard clinical interview. Clinical interviews were conducted within 24 hours of admission. Patients were included in the study only if both raters agreed on the diagnosis. Diagnosis of a manic episode was made according to ICD-10 clinical criteria. Patients with a diagnosis of mixed bipolar affective disorder were excluded. Patients were evaluated using the YMRS within 24 hours of admission by trainee psychiatrists trained in the use of the YMRS.

The YMRS is an eleven item clinician administered scale used to measure the severity of mania. Items include elevated mood, increased motor activity-energy, sexual interest, sleep, irritability, speech, language-thought disorder, thought content, disruptive aggressive behaviour, appearance and insight. In scoring the YMRS, four items (irritability, speech, thought content and disruptive/aggressive behaviour) are graded on a 0 to 8 scale. Seven items (elevated mood, increased motor activity/energy, sexual interest, sleep, language/thought disorder, appearance and insight) are graded on a 0 to 4 scale. There are no items measuring low mood. Ratings are based on a patient's subjective report of his or her condition over the previous 48 hours and the clinician's observations during the interview [14]. The score for each item is summed to obtain the total score for the scale. Higher scores reflect more severe level of psychopathology. Internal consistency as measured by Cronbach's alpha was 0.72.

Factor analysis is a statistical technique which defines the underlying structure among variables. Factor analysis of clinical features can be used to identify clinically meaningful subtypes of illness.

Exploratory factor analysis (principal component analysis) was carried out to identify factors. We chose principal component analysis as almost all the factor analytic studies of manic symptoms had used this method. Only factors with an eigenvalue > 1 were retained. This was compared with factors identified by examining a Cattell's scree plot. The significance level for interpretation of factor loadings was 0.40. The cut-off for determining the significance level was based on the sample size. Based on a significance level of 0.05 and a power of 80% a sample of 200 is required to consider a factor loading of 0.4 as significant [15]. A cut off of 0.4 has been used previously in studies of bipolar patients [16].

Items which loaded on more than one factor were included in the factor for which they had the highest factor-loading score. The unrotated component matrix identified five factors. Oblique rotation (Promax with Kaiser Normalization) was then carried out to identify three factors which were clinically meaningful. Statistical analysis was carried out using SPSS version 16.0.

## Results

### Sample description

The sample of 131 patients included 76 (58%) males. The age range was 16-79 years with a mean age of 44.05 years (SD = 15.6).

### Factor analysis

Examination of communalities showed all variables were within the accepted range except disruptive aggressive behaviour (0.483). This variable was retained because of its clinical importance. Kaiser-Meyer-Olkin measure of sampling adequacy was 0.522 and Barlett's test of sphericity was significant.

Table 1 shows the unrotated principal component analysis of the Young Mania Rating Scale items. Unrotated principal component analysis identified five factors with eigenvalues > 1. Factor one (15.4% of variance) had significant loadings for 3 variables: increased motor activity/energy, irritability and disruptive-aggressive behaviour. It had significant negative loadings for thought content and speech. Factor 2 (13.9% of variance) included elevated mood, language abnormalities/thought disorder (circumstantial, distractible, flight of ideas, incoherent) increased sexual interest and poor insight. Three items loaded on factor 3 (12.3% of variance): increased motor activity/energy, abnormalities in thought content (grandiose, paranoid ideas, ideas of reference, delusions and hallucinations) and appearance (impaired self-care). Factor 4 (12.2% of variance) included disruptive, aggressive behaviour and appearance (impaired self-care). It had a significant negative loading for increased sexual interest. Factor 5 (11.5% of variance) contained speech abnormalities (pressured speech) and insight and negative loading for sleep. Four variables (increased motor activity/energy, disruptive-aggressive behaviour, insight and appearance) loaded on two factors.

**Table 1:** Unrotated factor loadings of the Young Mania Rating Scale items in patients with acute mania

Variables	Factor 1	Factor 2	Factor 3	Factor 4	Factor 5
Irritability	.580	-.209	.235	.054	.294
Increased motor activity/energy	.706	-.158	.480	-.235	.122
Disruptive-aggressive behaviour	.476	.079	.196	.459	.029
Language-thought disorder	.359	.619	-.317	.170	-.053
Elevated mood	.392	.601	.347	-.172	.000
Insight	-.275	.496	-.191	.121	.488
Thought content	-.438	-.142	.636	-.200	-.147
Sexual interest	.034	.500	.041	-.672	-.080
Appearance	-.308	.182	.455	.545	.219
Speech (rate and amount)	-.438	.108	.240	-.191	.595
Sleep	-.238	.404	.379	.261	-.534

Table 2 shows factor loadings after oblique rotation (Promax with Kaiser Normalization). Factor 1corresponding to 'irritable mania' had significant loadings for three variables, irritability, increased motor activity/energy and disruptive aggressive behaviour. Factor 2 corresponding to 'elated mania' had significant loadings for four variables, elated mood, language abnormalities/thought disorder, increased sexual interest and poor insight. Factor 3 corresponding to 'psychotic mania' had significant loadings for four variables, abnormalities in thought content, appearance (impaired self-care), poor sleep and speech abnormalities.

**Table 2:** Principal component analysis, with rotation (Promax with Kaiser Normalization) of the Young Mania Rating Scale items in patients with acute mania

Variables	Factor 1	Factor 2	Factor 3
Irritability	.772	-.045	.093
Increased motor activity/energy	.736	-.064	-.189
Disruptive-aggressive behaviour	.474	.172	-.055
Language-thought disorder	-.031	.690	-.336
Elevated mood	.390	.663	.212
Sexual interest	-.052	.497	.110
Insight	-.430	.434	.064
Thought content	.060	-.246	.734
Appearance	-.011	.104	.574
Sleep	-.046	.338	.516
Speech (rate and amount)	-.225	.009	.442

There was little correlation between factors. Factor 3 was negatively correlated with factor 1 (-0.079) and factors 2 (-0.60). Correlation between factors 1 and 2 was 0.054

## Discussion

The factor analysis of manic symptoms identified using the YMRS found three clinically meaningful factors. These factors represent 'elated mania', 'irritable mania' and 'psychotic mania'.

The first factor represented irritable mania with high loadings from irritability, and increased motor activity/energy. Disruptive-aggressive behaviour also loaded on it. Significant negative loadings were seen for lack of insight, indicating that insight was retained in irritable mania in contrast to elated mania. The association of irritability with aggressive behaviour has been described previously [11,13].

Although our study found a clear separation of items loading on the two factors irritable mania and psychotic mania, two studies have described a single factor incorporating items of psychosis and irritable mania [4,8]. However most studies found two separate factors corresponding to 'psychotic mania' and 'irritable mania' [5,11,13,17,18].

The second factor represented elated mania. Four items which are considered classical manic symptoms i.e. elevated mood, language/thought disorder (racing thoughts, circumstantial, distractible, flight of ideas, incoherent), increased sexual interest and poor insight loaded on this factor. Thought content which had significant loading for psychotic mania, loaded negatively on this factor indicating that features of classical mania were separate from psychotic mania.

Most factor analytic studies identify elevated mood, hypersexuality and grandiosity as core features of mania. The scale we used, the YMRS does not have a separate item describing grandiosity. Instead it includes grandiose ideas along with psychotic features of delusions and hallucinations in the item named 'thought content'. Therefore in our study the item including grandiosity did not load on this factor. Features which are commonly associated with mania such as poor sleep, increased motor activity and pressured speech did not load on this factor either. This has been reported in other studies too [7,10,11,17]. Sato et al and Rossi et al. describe poor sleep as a separate factor [10,11]. Gupta et al. report the items pressured speech and racing thoughts/disturbed concentration loading as a separate factor which was named 'accelerated thought stream' [17].

The third factor represented psychotic mania. It has significant loadings for four items: abnormalities in thought content, appearance (impaired self-care), poor sleep and speech abnormalities (rate and amount). The highest loadings were from 'thought content'. In the YMRS, most psychotic phenomena are included in the item 'thought content' which describes grandiose and paranoid ideas, ideas of reference, delusions and hallucinations. The item appearance (describing impaired self-care) also loaded on this factor which suggests the possibility of functional deterioration associated with psychotic mania. Psychotic mania is conceptualized as a more severe form of mania and it is associated with poorer levels of social functioning [19,20].

Our study supports findings from other studies which suggest a multidimensional structure of mania [5,8-13,17]. There does not appear to be clear consensus about the factor structure of manic episodes. The use of different scales limit the comparison of findings between studies because the number of factors identified and the items loading on factors depend on the structure of the scales. Despite this, most studies have identified factors corresponding to classical mania, irritable mania and psychotic mania [5,9,11,17,18].

Although our findings support a multidimensional phenomenological model of mania, the evidence to support distinct clinical subtypes is still inadequate. Psychotic mania has been described as a separate factor by several authors [5,9,17,18]. It is already included in the ICD-10 as a subtype (mania with psychotic symptoms) and the DSM-IV recognises psychotic features as an indication of the severity of illness.

One of the most significant findings of this factor analysis is the identification of a factor corresponding to irritable mania. Neither ICD-10 nor DSM-IV diagnostic systems recognize 'irritable mania' as a distinct category. Evidence supporting a distinct clinical subtype of irritable mania is still scanty. A study reports that patients with irritable mania have higher psychomotor depression, irritability, later onset, and lower episode density compared to other subtypes [13]. Since there is adequate evidence to support irritable mania as a phenomenological category, evidence from family history, response to treatment, clinical course and stability of symptoms across different episodes must be sought to support the existence of a clinical subtype.

There are several limitations in our study. Although there is evidence from other studies about the existence of a depressive factor in patients with mania, we did not identify a depressive factor [5,8-11,13,17]. This is because of the non-inclusion of depressive items in the scale we used and because we did not include patients with mixed mania. Our study was a cross sectional study which considered symptoms on admission only. Diagnosis was made following a standard clinical interview and not a structured interview designed for research purposes. We are unable to identify the stability of the subcategories over several episodes. We did not attempt to identify associations between the factors and demographic and clinical characteristics. We did not perform a cross-validation to double check the present pattern of results.

## Conclusions

Our findings identified three clinically meaningful factors corresponding to 'elated mania',' irritable mania' and 'psychotic mania'. These findings support the multidimensional nature of manic symptoms. Further evidence is needed to support the existence of corresponding clinical subtypes.

## Competing interests

The authors declare that they have no competing interests.

## Authors' contributions

Both authors contributed to designing the study, supervision of data collection, analyzing the data and drafting the manuscript. Both authors read and approved the final manuscript.

## Pre-publication history

The pre-publication history for this paper can be accessed here:

http://www.biomedcentral.com/1471-244X/11/137/prepub
